# Supplementary material for: STAT5B leukemic mutations, altering SH2 tyrosine 665, have opposing impacts on immune gene programs
Source: bioRxiv. 2024 Dec 22:2024.12.20.629685. Preprint. [Version 1] doi: 10.1101/2024.12.20.629685 (PMC11722272; doi:10.1101/2024.12.20.629685)
Supplement: 1 [file NIHPP2024.12.20.629685v1-supplement-1.pdf]

bioRxiv preprint doi: https://doi.org/10.1101/2024.12.20.629685; this version posted December 22, 2024. The copyright holder for this preprint (which was not certified by peer review) is the author/funder. This article is a US Government work. It is not subject to copyright under 17 USC 105 and is also made available for use under a CC0 license.

Supplementary Figures

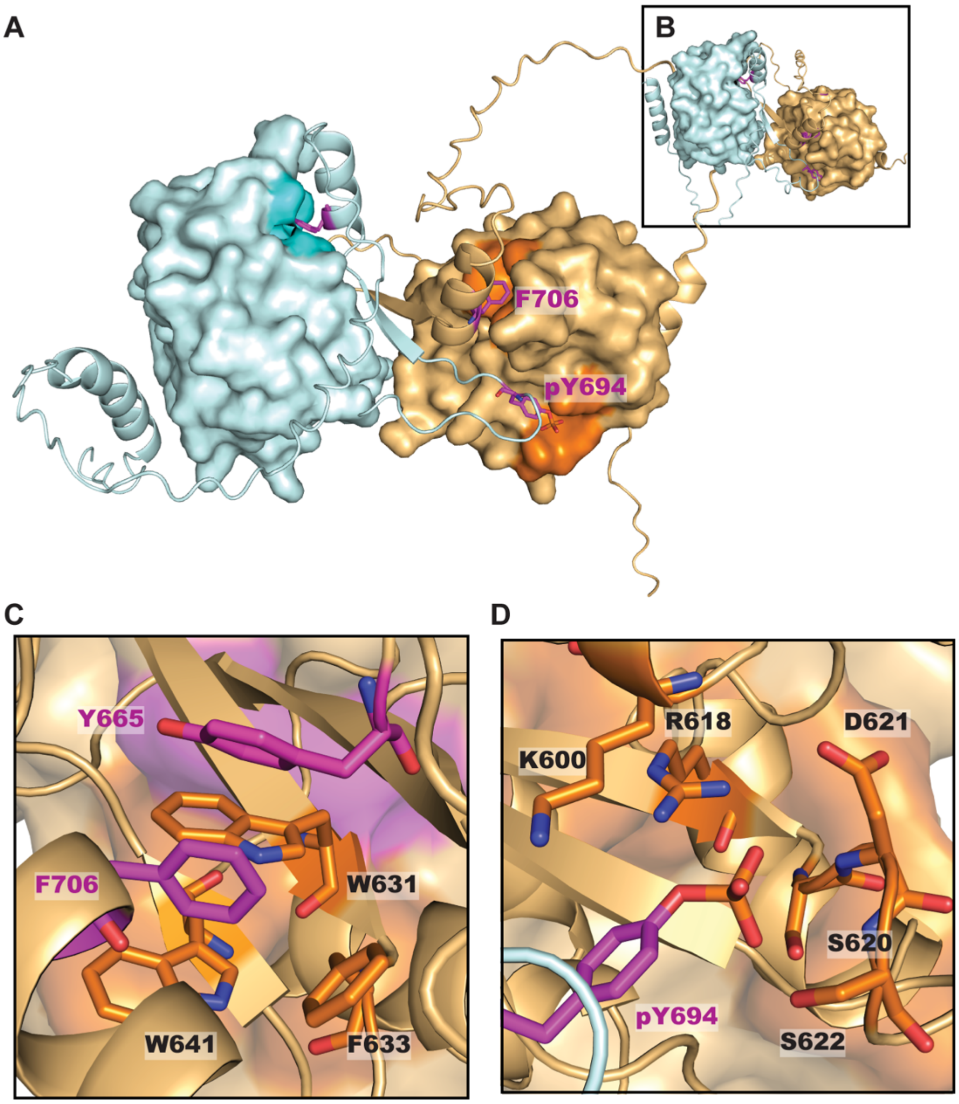

Supplementary Fig. 1. STAT5 SH2 dimerization modeled by AlphaFold3. A Structure of the human STAT5A SH2 homodimer generated by AlphaFold3. Binding pockets of key residues phospho-Tyr694 and Phe706 (purple), which are structurally analogous to phosphor-Tyr699 and Phe711 in STAT5B, are indicated. **B** Structure of the human STAT5B SH2 homodimer generated by AlphaFold3, which closely resembles the dimer interface modeled for the STAT5A homodimer. **C** The STAT5A model highlights the intramolecular interaction between Phe706 (purple) and the hydrophobic binding pocket (orange) containing key residue Tyr665 (purple). **D** The STAT5A model highlights the canonical SH2 docking interaction between the phospho-Tyr694 (purple) of the STAT5A C-terminal tail and the canonical SH2 binding pocket of a second STAT5A (orange).

bioRxiv preprint doi: https://doi.org/10.1101/2024.12.20.629685; this version posted December 22, 2024. The copyright holder for this preprint (which was not certified by peer review) is the author/funder. This article is a US Government work. It is not subject to copyright under 17 USC 105 and is also made available for use under a CC0 license.

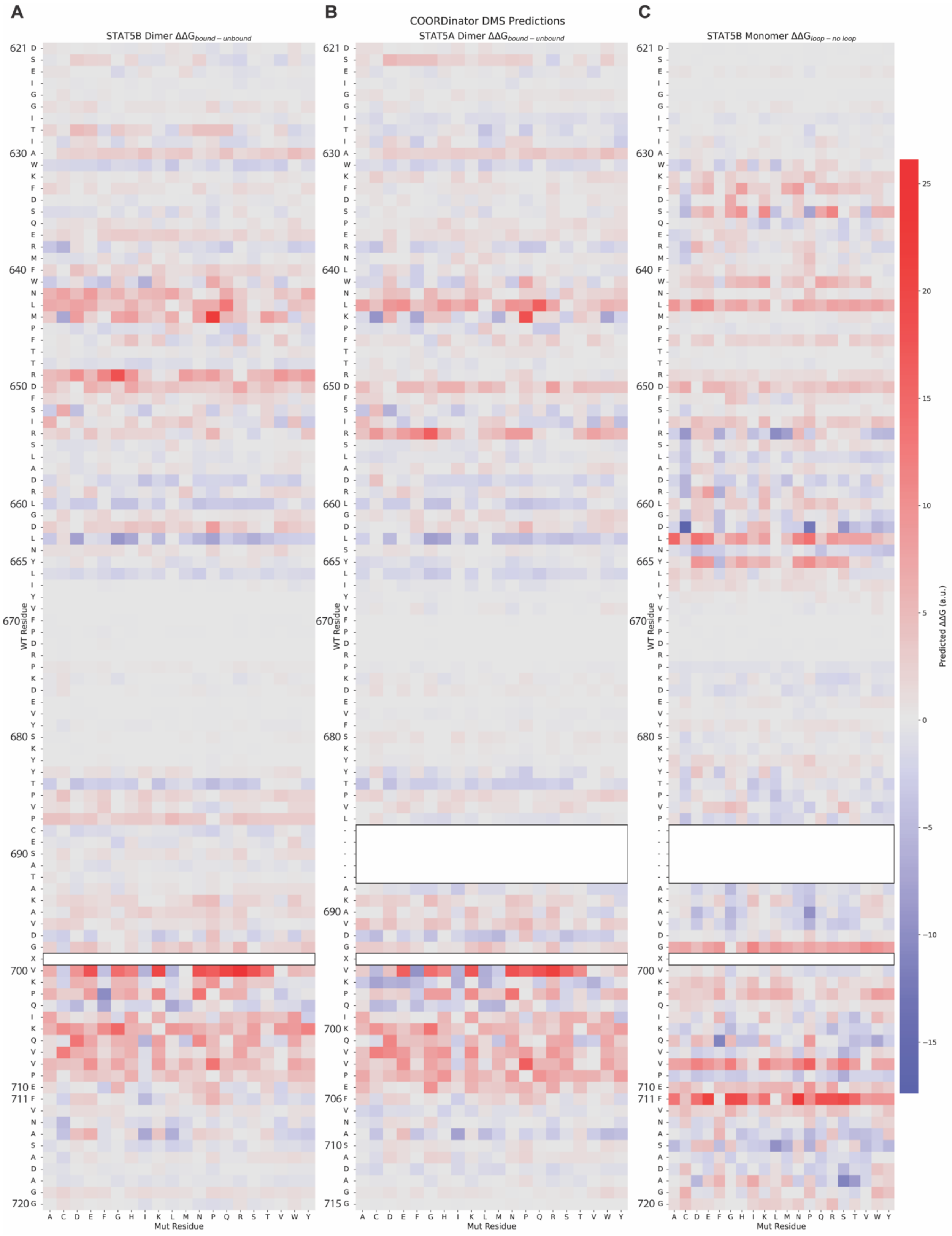

bioRxiv preprint doi: https://doi.org/10.1101/2024.12.20.629685; this version posted December 22, 2024. The copyright holder for this preprint (which was not certified by peer review) is the author/funder. This article is a US Government work. It is not subject to copyright under 17 USC 105 and is also made available for use under a CC0 license.

Supplementary Fig. 2. Energetic contributions of STAT5 SH2 domain residues to dimerization and intra-molecular interactions as predicted by COORDinator. Heat maps depicting the energetic consequences of amino-acid substitutions at each residue as predicted by COORDinator using AlphaFold3-generated structures. Relative mutational effects are annotated using arbitrary units with stabilizing mutations ($-\Delta\Delta G$) depicted in blue and destabilizing mutations depicted in red ($+\Delta\Delta G$). The intensity of the color corresponds to the extent of the change. Values for $STAT5A^{pY694}$ and $STAT5B^{pY699}$ are not included due to the inability of COORDinator to model post-translational modifications. **A** Energetic contributions of STAT5B residues to SH2 homodimerization. **B** Energetic contributions of STAT5A residues to SH2 homodimerization. Residues 688-692 are not present in STAT5A. **C** Energetic contributions of STAT5B residues to interaction with the C-terminal tail, when the tail is modeled as a separate chain. Residues 688-692 were not modeled to ensure the tail is properly seen by the model as a separate chain.

bioRxiv preprint doi: https://doi.org/10.1101/2024.12.20.629685; this version posted December 22, 2024. The copyright holder for this preprint (which was not certified by peer review) is the author/funder. This article is a US Government work. It is not subject to copyright under 17 USC 105 and is also made available for use under a CC0 license.

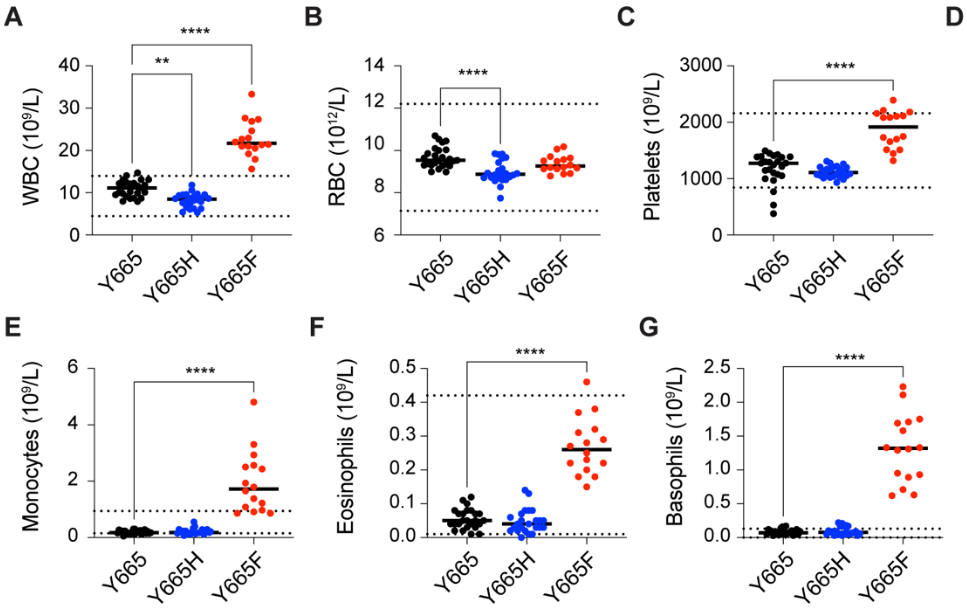

Supplementary Fig. 3. Hematological parameters and altered immune phenotypes of Stat5b mutant mice. Count of Blood Cells in peripheral blood from 7-10-week-old adult wild-type and mutant mice. Results are shown as the median of independent biological replicates (Y665, *n* = 25; Y665H, *n* = 24; Y665F, *n* = 16). *P*-value are from two-way ANOVA with Tukey's multiple comparisons test. **P* < 0.05, ***P* < 0.01, ****P* < 0.0001, *****P* < 0.0001.

bioRxiv preprint doi: https://doi.org/10.1101/2024.12.20.629685; this version posted December 22, 2024. The copyright holder for this preprint (which was not certified by peer review) is the author/funder. This article is a US Government work. It is not subject to copyright under 17 USC 105 and is also made available for use under a CC0 license.

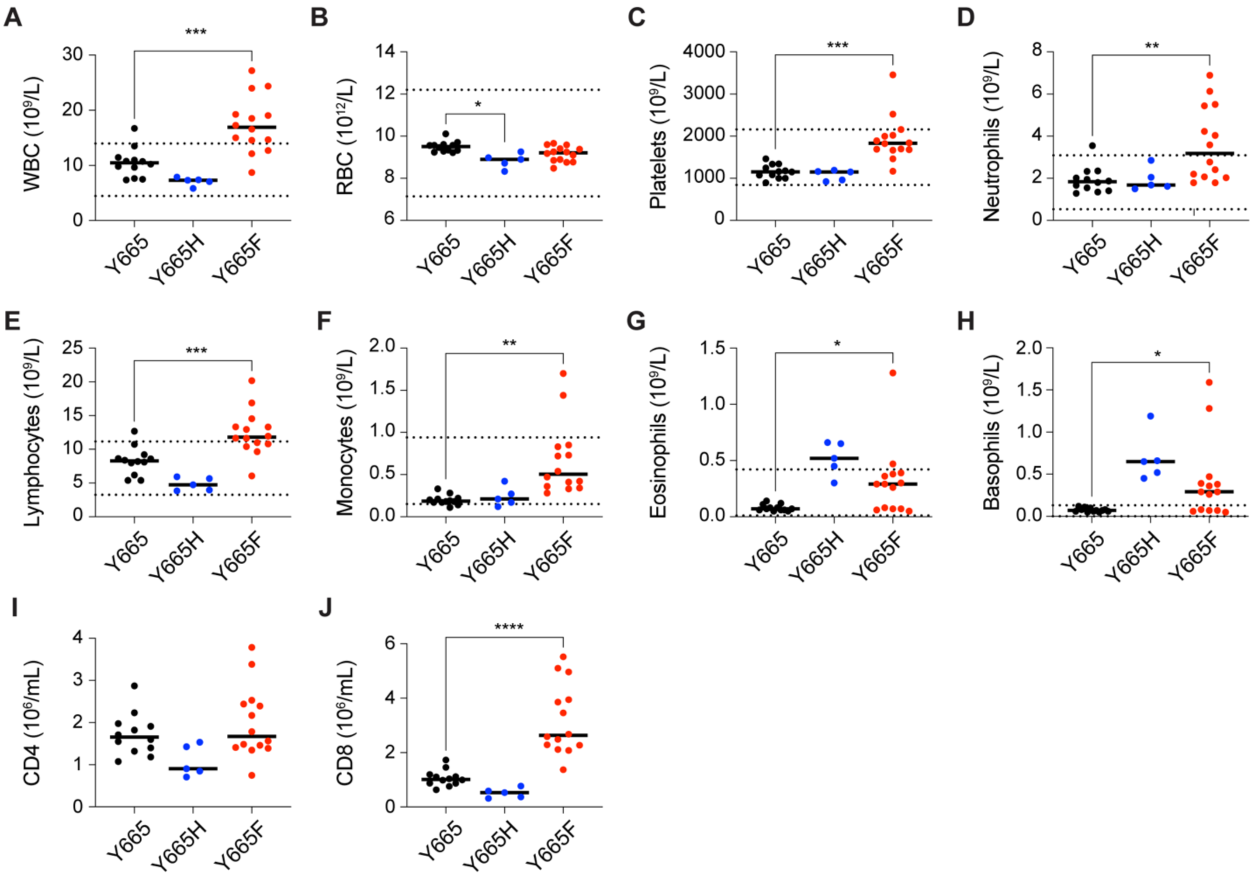

Supplementary Fig. 4. Hematological parameters and altered immune phenotypes of Stat5b mutant mice. A-H Count of Blood Cells in peripheral blood from 11 months-old adult wild-type and mutant mice. Results are shown as the median of independent biological replicates (Y665, *n* = 12; Y665H, *n* = 5; Y665F, *n* = 14). **I-J** Numbers of subpopulation of immune cells identified by flow cytometry. Results are shown as the median of independent biological replicates (*n* = 5). Statistical significance was assessed using one-way ANOVA followed by Tukey's multiple comparisons test. **n** Apoptosis rate detected by Annexin V and 7-amino-actinomycine staining using flow cytometry. *P*-value are from two-way ANOVA with Tukey's multiple comparisons test. **P* < 0.05, ***P* < 0.01, ****P* < 0.0001, *****P* < 0.0001.

bioRxiv preprint doi: https://doi.org/10.1101/2024.12.20.629685; this version posted December 22, 2024. The copyright holder for this preprint (which was not certified by peer review) is the author/funder. This article is a US Government work. It is not subject to copyright under 17 USC 105 and is also made available for use under a CC0 license.

Supplementary Tables

Supplementary Table 1. List of genes that are significantly up-regulated by Stat5b mutant plasmids in CD4+ T cells from STAT5-deficient mice.

Supplementary Table 2. List of significantly up-regulated genes in IL-2/7 stimulated T cells from spleen of STAT5B^{Y665F} and STAT5B^{Y665H} compared to IL-2/7 stimulated T cells from Y665 and unstimulated T cells from STAT5B^{Y665F} with normalized read counts at spleen tissue, log2 (fold change), their *p*-value and adjusted *p*-value. List of genes with or without STAT5 binding on their regulatory elements.

Supplementary Table 3. List of significantly up-regulated genes in STAT5B^{Y665F} compared to STAT5B^{Y665H} with normalized read counts at spleen tissue, log2 (fold change), their *p*-value and adjusted *p*-value. List of genes with or without STAT5 binding on their regulatory elements.

Supplementary Table 4. List of STAT5B binding peaks, STAT5B bound enhancers and enhancer clusters in STAT5B^{Y665H} and STAT5B^{Y665F} mice.

Supplementary Table 5. Sequences of sgRNA for CRISPR/Cas9 and base-editing targeted mice. The donor oligo is contained the desired Y (TAC) to F (TTT) change.

Target site	sgRNA sequences
STAT5B^{Y665H}	5′-TGAGGTAATTCAGGTCCCCCAGG-3′
STAT5B^{Y665F}	5′-TGAGGTAATTCAGGTCCCCCAGG-3′<br><br>Donor Oligos<br>GGAATCTGATGCCTTTTACCACTAGAGACTTCTCTATCCGGTCCCTCGCTGA<br>CCGCCTGGGGGACCTGAATTTTCTCATATATGTGTTTCCTGATCGGCCAAAG<br>GATGAAGTATATTCTAAGTACTACACACC
STAT5A^{Y668H}	5′-GGTCTGGGAACACAAAAATAAGG-3′
STAT5A^{Y668F}	5′-GGTCTGGGAACACATGAATAAGG-3′
